# A Review of the Effect of a Nanostructured Thin Film Formed by Titanium Carbide and Titanium Oxides Clustered around Carbon in Graphitic Form on Osseointegration

**DOI:** 10.3390/nano10061233

**Published:** 2020-06-24

**Authors:** Roberto Scandurra, Anna Scotto d’Abusco, Giovanni Longo

**Affiliations:** 1 Department of Biochemical Sciences, Sapienza University of Roma, Piazzale A. Moro 5, 00185 Roma, Italy; anna.scottodabusco@uniroma1.it; 2 Consiglio Nazionale delle Ricerche-Istituto di Struttura della Materia, Via del Fosso del Cavaliere, 00133 Roma, Italy; longo@ism.cnr.it

**Keywords:** titanium, titanium carbide, Pulsed Laser Deposition, Ion Plating Plasma Assisted, TiC coating, graphitic carbon, thin film, biocompatibility, implant osseointegration, human primary osteoblasts

## Abstract

Improving the biocompatibility of implants is an extremely important step towards improving their quality. In this review, we recount the technological and biological process for coating implants with thin films enriched in titanium carbide (TiC), which provide improved cell growth and osseointegration. At first, we discuss the use of a Pulsed Laser Ablation Deposition, which produced films with a good biocompatibility, cellular stimulation and osseointegration. We then describe how Ion Plating Plasma Assisted technology could be used to produce a nanostructured layer composed by graphitic carbon, whose biocompatibility is enhanced by titanium oxides and titanium carbide. In both cases, the nanostructured coating was compact and strongly bound to the bulk titanium, thus particularly useful to protect implants from the harsh oxidizing environment of biological tissues. The morphology and chemistry of the nanostructured coating were particularly desirable for osteoblasts, resulting in improved proliferation and differentiation. The cellular adhesion to the TiC-coated substrates was much stronger than to uncoated surfaces, and the number of philopodia and lamellipodia developed by the cells grown on the TiC-coated samples was higher. Finally, tests performed on rabbits confirmed in vivo that the osseointegration process of the TiC-coated implants is more efficient than that of uncoated titanium implants.

## 1. Introduction

Dental and orthopedic prostheses are made of titanium, a light, strong and inexpensive metal. Unfortunately, while extremely useful, titanium is also one of the most oxidizable elements in nature, and it reacts spontaneously with the oxygen in air to produce titanium oxides, mainly titanium dioxide (TiO_2_), forming a very thin layer (less than 10 nm) [1,2,3,4]. This TiO_2_ can be found in two phases, anatase and rutile, and has known catalytic properties, which could be involved in its interaction with the bodily fluids. This passivated layer made of titanium oxides is considered to confer to titanium implant its high biocompatibility, making this one of the most used materials for biological uses [5,6]. However, this oxide layer may grow in the harsh conditions of biological fluids, and become a non-metal layer interposed between the metal of the implant and the bone. Osteoblasts should then approach this passivating layer, which does not form good chemical bonds with bone, is more brittle than metal, and may form fractures from which titanium oxide nanoparticles may be produced. It can also attract macrophages and neutrophiles that, releasing cytokines, attract fibroblasts, instead of osteoblasts, driving the production of a fibrotic tissue around the implant instead of a bone tissue [7,8,9].

The chemical composition of the titanium oxide layers is well-known to induce the production of a large number of small adhesion areas, and this can lead to fibrinogenesis, soft tissue encapsulation of the non-biological substrate, micromotion of the implant, loosening and failure [10,11,12]. This is unfortunately quite a common outcome for implants, and causes distress and large social costs. In addition, the titanium implants exposed to such a harsh environment can form titanium nanoparticles, which, due to their nanoscale, are easily interiorized by the adhering cells, inducing toxic reactions and causing further implant loosening [13].

In order to avoid these drawbacks, coating titanium implants with a protective layer using various technologies is one of the more common strategies. Among these, many physical and chemical modifications of the implant surfaces have been proposed, such as coating with titanium dioxide or titanium nitride [14,15]. Other works have described the coating of titanium with various ceramic materials, deposited using a wide range of techniques such as Physical Vapor Deposition (PVD), Chemical Vapor Deposition (CVD) or Plasma Spray Electrolysis (PSE) [16,17,18]. Since bone tissue is formed of about 60% hydroxyapatite, coating titanium implants with this material has been attempted, in order to protect the implant and at the same time increase its biocompatibility [18,19]. However, while hydroxyapatite provides some osseoinductive properties, its modest mechanical properties and brittleness may induce the formation of particles, which, similarly to the titanium ones, can cause inflammation and may lead to the formation of a fibrotic tissue and implant loosening, till its rejection from the body [3,11]. Other works have proposed different coating techniques and various layer compositions to enhance the properties of the layer. For instance, in a very recent work, Xia et al. detailed how plasma immersion implantation can be used to embed C/Cu ions in the implant surface, in order to provide added bactericidal and osseoinducing properties to the implants [20].

Another common strategy for enhancing the implant’s integration and cellular adhesion is to coat the implant’s surface with biomimetic molecules. Liu et al. obtained very encouraging results by adhering short peptide chains on substrates, such as adhesion proteins, i.e., fibronectin [19]. This strategy has the advantage of involving natural pathways and proteins commonly found in vivo; however, it presents several drawbacks, such as the high costs of the peptides and the difficulty in defining a protocol to achieve robust protein adhesion to implant surface. Particularly interesting are the approaches to osseointegration that involve Guided Bone regeneration, in which different materials are used to produce a localized bone formation for optimal bone reconstruction. In a recent example, Zhang and coworkers used MXenes, two-dimensional highly biocompatible materials, as membranes to guide and enhance the bone formation in dental implants [21].

Several works have detailed techniques to coat titanium implants with a protective layer, which shields the implant from oxidation while at the same time providing additional useful properties to the surface, such as improved hardness, controlled micro and nano roughness, and good wettability, whilst also promoting stimulating effects on osteoblasts growth [22,23]. These characterizations, at both the surface science and the biological level, require the combination of a large number of techniques. For instance, the combination of Atomic Force Microscopy (AFM) and Scanning Electron Microscopy (SEM) is widely employed to characterize surface structures and the evolution of biological systems in different conditions [24]. AFM in particular was born as a pure morphological characterization tool, but has become in the last 20 years a versatile investigation system, for studying mechanical properties at the nanoscale [25,26,27,28], single molecule elasticity and characteristics [29,30], and cellular morphology, behavior and interactions [31], especially coupled with high-resolution spectroscopic tools [32], and it has led to more interesting applications in the field of nanosensors [33,34,35,36].

Another technique, Transmission Electron Microscope (TEM), is a more complex surface investigation tool which can be coupled with other characterization and manipulation instruments, such as the Selected Area Electron Diffractometer (SAED), for the analysis of the crystalline structure of the sample, the Energy Dispersion Spectrometer (EDS), for chemical analysis, and the ion-beam columns Field Emission Gun (FEG), for micro and nano sample manufacturing [37,38,39].

Regarding the chemical composition evaluation of a surface, while X-ray Photoelectron Spectroscopy (XPS) is the technique of choice to study the chemical composition of surfaces, Raman spectroscopy allows a more detailed view of the elemental interactions, highlighting at the molecular level the surface composition [40,41,42].

## 2. TiC Layers Obtained Using Pulsed Laser and Ion Plated Plasma Assisted Deposition: A Review of Their Physical, Mechanical and Biological Properties

Recent works have identified titanium carbide (TiC) as a promising coating material, which can be deposited on titanium implants and prostheses, providing a protection to the underlying bulk material and an improved hardness and controlled micro and nano roughness, with, in addition, an excellent biocompatibility. For instance, Kumar and coworkers used magnetron sputtering to coat stainless steel surfaces with TiC and ZrC layers to improve biocompatibility. These carbides favor the adhesion of artificial plasma proteins, which induce a faster and more reliable osseointegration. This work also monitored how these coatings influence bacterial adhesion, which is one of the possible causes of implant rejection [43]. Similarly, in Olah et al., a TiC layer was produced using sputtering on different substrates, and an in-depth characterization of the structural, mechanical and electrochemical properties of the layer was performed, showing that the TiC layer provided a longer lifetime of implants in the body [44]. Finally, Kao and coworkers detailed how treating the substrates before TiC coating could produce a more uniform deposition with better wear resistance, anti-corrosion properties, and biocompatibility performances [45].

Here, we point to two techniques that have been used in the last 10 years to produce these TiC-rich layers—the Pulsed Laser deposition and the Ion Plated Plasma Assisted deposition—while providing a full characterization of the chemical and physical properties of the layers. We also describe the in vitro and in vivo experiments that were performed, to demonstrate the effect of the TiC layers on osteoblast growth and the improved and faster osseointegration of implants.

### 2.1. Pulsed Laser Deposition

**Figure 1:**
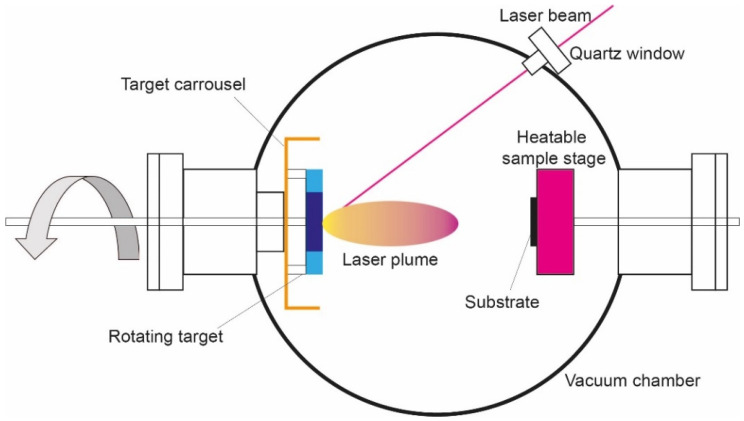
Schematics of a Pulsed Laser Deposition (PLAD) apparatus present at the Department of Physics of the Sapienza Rome University. When a solid target is irradiated by a focalized pulsed laser beam, a gaseous cloud, known as a plume due to its shape, is produced. The plume, a plasma composed of electrons, atoms, ions, molecules, clusters and, in some cases, droplets and target fragments, expands in vacuum, and will be deposited on the substrate, giving rise to a film where fragments of the target (spalls) may be inserted.

In Pulsed Laser Deposition (PLAD) (Figure 1), a focalized pulsed laser beam oriented with an inclination angle of 45°, produced by a Nd:Yag laser source (λ = 532 nm; τ = 10 ns; repetition rate 10 Hz), hits a titanium carbide (TiC) target with a laser fluence of 10 J/cm^2^, for a deposition time of 1 h, forming a gaseous cloud (the plume). This is formed by a plasma which is a mixture of atoms, ions, molecules, clusters, droplets and target fragments. It deposits on the titanium substrate, forming a film bearing small TiC fragments (spalls). In addition to the laser fluence, the number of spalls depends on the distance of the sample from the target. In our experimental conditions we determined that the best biological results were obtained by placing the titanium sample (disk or dental screw, the latter continuously rotated) 8 mm from the target [22,46,47,48].

It is widely reported that the morphology of the surface, specifically the micro and nanoroughness, is a factor that stimulates osteoblast growth [12,49,50,51,52]. Typically, this is obtained by imposing an artificial roughness on the substrates, by blasting them with zirconia particles. In addition, the deposition with the PLAD technology, using a laser fluence of 9–13 J/cm^2^, formed spalls in the TiC layer, producing an additional roughness [22].

Brama et al. performed SEM imaging of the TiC layers, showing that the substrate surface appeared as a 2–3 µm thick homogeneous layer with a large number of spalls. XPS indicated a chemical composition of 20% TiC and 80% titanium oxides, which were distributed as T_2_O_3_ (11–13%); TiO_2_ (51–56%); and TiO (14–15%). Finally, using a nanoindenter, the mechanical properties of the layer were determined, showing a hardness of 10 GPa [22].

#### 2.1.1. Effects of the PLAD Layer on Osteoblast Homeostasis

Thymidine incorporation is the assay used to determine the cellular proliferation [53], and the MTT test, [3-(4,5-dimethylthiazol-2-yl)-2,5-diphenyltetrazolium bromide] based colorimetric assay, is used to evaluate cell viability and surface toxicity [54,55]. This showed that the layer did not induce any toxicity and, in fact, demonstrated the clear positive effect of the TiC coating on the osteoblast proliferation. Indeed, by combining SEM and AFM imaging, Brama and coworkers provided a complete morphological overview of the cellular growth, and phenotype using primary human osteoblasts and osteoblast cell lines (ROS-SMER#14 and hFOB1.19). The images showed that the cells on TiC appeared firmly spread on the substrate, bearing many more filopodia, compared to the cells on Ti, further supporting the positive effect of the TiC layer on osteoblast spreading [22].

The determination of improved osseointegration involves the evaluation of the expression of the genes involved in bone turnover: ALkaline Phosphatase (ALP), Collagen1A2 (COLL1A2), Osteocalcin (OC), Bone Morphogenetic Protein-4 (BMP-4), Core binding factor -1/osteoblast specific factor (Cbfa1/osf/2) and Tumor Growth Factor-β (TGFβ). Semiquantitative Polymerase Chain Reaction (PCR) and Quantitative-Real-Time PCR (q-RT-PCR) are the techniques of choice for these analyses [56].

In the case of PLAD layers, Brama et al. showed upregulation of all these genes in the cells grown on TiC-coated titanium disks, compared to the uncoated ones. Additionally, the measurement of InterLeukin-6 and Macrophage Colony Stimulating Factor expression in the cells cultured on TiC-coated substrates showed no measurable alteration of osteoclastogenesis, and osteoclast activity produced by cell–cell interaction and paracrine stimulation, compared to the uncoated ones [22].

#### 2.1.2. In Vivo Studies on the PLAD Layer

To determine the effect of a live biological environment on an implant, and to determine if indeed the treatment leads to an improved osseointegration, the final step is to evaluate this in vivo. Due to the ethical requirements of these particular experiments, all techniques that allow us to reduce the animal suffering or the number of employed animals must be preferred. In this sense, X-ray mammography can be used to evaluate the bone density formation in small animals, such as rabbits, without requiring the sacrifice of the animals at every experimental step. This was used by Brama et al. to determine in vivo the improved performances of the PLAD TiC film, implanting small TiC-coated dental implants in the femurs of rabbits [22]. The bone density around the implants was evaluated after 4 and 8 weeks using X-ray mammography. At the first time-point, this analysis demonstrated an increase in the bone density around the TiC-coated implant compared to the untreated ones. After 8 weeks from the implantation, the bone around both the uncoated and the TiC-coated implant was increased, but the coated implant evidenced greater bone formation.

**Figure 2:**
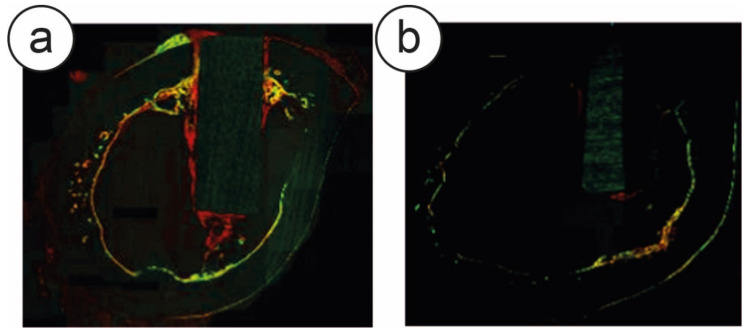
Confocal microscopy of intravitally stained small rods (2 × 5 mm) inserted in the femurs of rabbits. Stains indicate bone formation at 1 week (green), 2 weeks (red), 5 weeks (yellow). Significant bone was observed to have accumulated at the implant-bone interface, shown as red staining, at the 2-week time period near the TiC-coated implants and especially in the cancellous zone of the bone. Indeed, a significant quantity of new bone had already formed at 1 week near the TiC-coated implants [panel (**a**)]. Conversely, only a small amount of bone had formed around the uncoated Ti implants at 2 weeks [panel (**b**)] and no bone formation was visible at 1 week. Reprinted from [22]. Copyright (2007), with permission from Elsevier.

Differential fluorescent staining is an excellent way to add to the bone-density evaluation a time resolution, in order to monitor the bone growth and the osseointegration of the implants over time. Different fluorophores, administered at different time-points, are incorporated into new bone growth, therefore indicating the position of growing bone at the time of injection. Brama et al. performed these analyses and showed the rapid onset of bone formation around the TiC-coated rods, compared to the uncoated Ti ones (Figure 2) [22].

The overall results of the in vivo experiments strongly suggest that the coating of titanium implants with TiC-enriched film could be a very useful addition to the titanium dental and orthopedic implant production process, leading to an improvement in their success rate. Unfortunately, a practical application of the PLAD deposition process to orthopedic and dental implants would be far too complex for industrial applications, since the PLAD is not easily adaptable for the coating of more than one sample per cycle.

In order to overcome this limitation, whilst maintaining the advantageous properties of TiC-enriched film, Scandurra’s group proposed a second deposition procedure which can coat several samples and three dimensional implants in a single step, producing a well-controlled film: the Ion Plating Plasma Assisted (IPPA) with reactive magnetron sputtering [57,58].

### 2.2. Ion Plating Plasma Assisted Deposition

**Figure 3:**
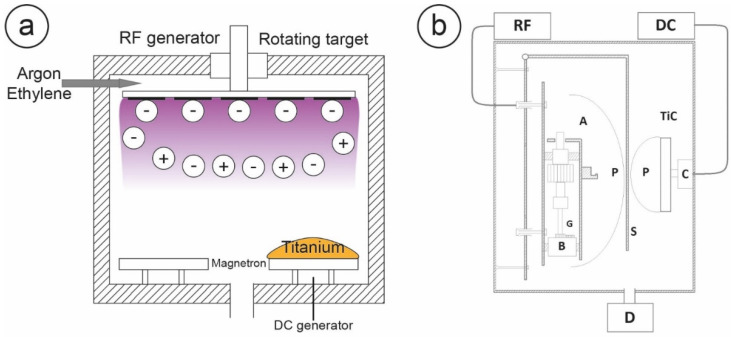
Panel (**a**): A general schematization of the Ion Plating Plasma Assisted (IPPA) apparatus. The sample holder containing the substrates to be treated is biased by a radiofrequency (RF) produced by the generator, while the magnetron sputtering device, on which a titanium target is placed, is powered by the Direct Current (DC) generator. Vacuum is applied to the chamber, followed by introduction of ethylene as carbon source: maintaining active both DC and RF, a plasma cloud is generated and produces the deposition on the substrates. Panel (**b**): the IPPA apparatus with the TiC target on the magnetron sputtering source, powered by variable direct current. The sample holder (A) containing the substrates biased by a constant RF produced by the generator, while the magnetron sputtering source (C) on which a TiC target is placed is powered by variable DC. Vacuum is applied to the chamber through the pump (D). Plasma clouds (P) are generated in front of the TiC target and the sample holder, and deposition is produced on the substrates. Reprinted from [63]. Copyright (2015), with permission from Elsevier.

The Ion Plating Plasma Assisted machine is an evolution of the Ion Plating procedure, which was originally proposed by Mattox and subsequently revived by Misiano [59,60,61]. As shown in Figure 3a, an IPPA apparatus is composed of a titanium target with a magnetron sputter source, which can produce titanium ions activated by a direct current (DC). The vacuum chamber is flushed with ethylene and argon, with the first serving as carbon source. Some of the ionized titanium is expelled by the magnetron source, and then accelerated by a negative polarization produced by a radiofrequency electric field (RF) applied to the sample holder. This also produces a plasma that contributes to generating a further polarization of the neutral titanium particles, inducing an ionic bombardment of the argon and carbon gas mixture on the growing film.

The protocol was defined and optimized by Longo et al. and Mazzola et al., who demonstrated an improved efficiency in the osseointegration of this coating, and fully characterized the morphological and chemical properties of the layer [57,58].

Mazzola et al. demonstrated, using XPS analysis, an increase in the amount of TiC (36%) with respect to that produced by coating using PLAD (20%), and a decrease in titanium oxides (63% in the IPPA layer against the 80% in the PLAD layer) [58]. Measures of nanoindentation demonstrated that the film had a thickness of about 400 nm, strongly adherent to the bulk titanium, with a strength of 25–30 GPa, two to three times that found in the films deposited by PLAD and a high elastic modulus (282 GPa). The AFM imaging showed that the surface of the film deposited by IPPA was uniform in all areas, and in comparison with the surface of uncoated titanium, the film had introduced only minor morphological differences. Longo et al. combined this with AFM and SEM, which evidenced that osteoblasts reacted to the morphology and chemistry of the layer, producing more filopodia and better adhering to the substrates as compared to untreated surfaces, even at very short incubation times (6 h). Finally, this effect was monitored by evaluating the increase in gene expression of proteins involved in bone turnover [57].

Subsequent optimization procedures evidenced how an IPPA deposition chamber, with a titanium carbide target as a simultaneous source of titanium and carbon (Figure 3b), could provide a more uniform coating, maintaining the cellular stimulation and underlying the implant protection properties of the layer. This is similar to what was done in the PLAD apparatus, but with the reliability of the IPPA technology, and an easy scalability to the industrial level.

It is worth noting that the energization around the TiC target of the condensing material by a laser beam, as in PLAD, or by a DC-activated magnetron as in IPPA, and the bombardment of the growing film with energetic particles, induce a very similar deposition. Both techniques deposit layers through neutral particles, and accelerate a plasma formed by various elements which surround the substrate. A part of the TiC is deposited on the Ti substrate, while another part is dissociated in the reactive ions Ti^+^ and C^−^ by the ion bombardment and by contact with the plasma. This modelization of the deposition implies also that the C^−^ ions can either directly react near the substrate surface, or that the C_2_ molecules can condense due to the high temperature of the plasma clouds, to form graphitic rings [62]. These are thermodynamically favored forms, and these rings can produce clusters of graphitic carbon which are then found inside the nanostructure as a consequence of the deposition process. In addition, since the affinity of Ti for oxygen is extremely high (oxygen equilibrium pressures over Ti*_x_*O*_y_* are around 10^−37^ Torr at the temperature of 1000 K) [3], at the vacuum conditions typical of PLAD and IPPA (10^−8^ Torr), even the small partial oxygen pressure was sufficient to produce many titanium oxides. The Ti^+^ ions present on the substrate surface easily react with residual oxygen to produce TiO, TiO_2_ and Ti_2_O_3_, all found through Ti p2 XPS spectra [63]. The structural and the chemical composition of the film deposited by IPPA is thus very similar to that of the PLAD film, with the only major difference being in their respective compactness, which is higher in the IPPA film.

#### 2.2.1. IPPA Layer Characterization and Optimization

The IPPA deposition involves a large number of different deposition parameters that can be changed to improve the biocompatibility and the integration efficiency of the TiC layer. In the case of osteoblasts, the best deposition setup must be determined by monitoring the expression of ALP, COLL 1A2 and OC, the three protein genes involved in the bone turnover. The deposition method can be optimized by fixing the radiofrequency applied to the sample holder and varying the power applied to the magnetron source, evaluating the variation of the resulting protein gene expression compared to the uncoated substrates. Zanoni et al. showed how 900 Watt of DC applied at the magnetron source, and 100 W radiofrequency at the sample holder, and a deposition time of 30 min to reach a coating thickness of about 500 nm, were optimal deposition conditions to produce an ideal cellular growth [63].

**Figure 4:**
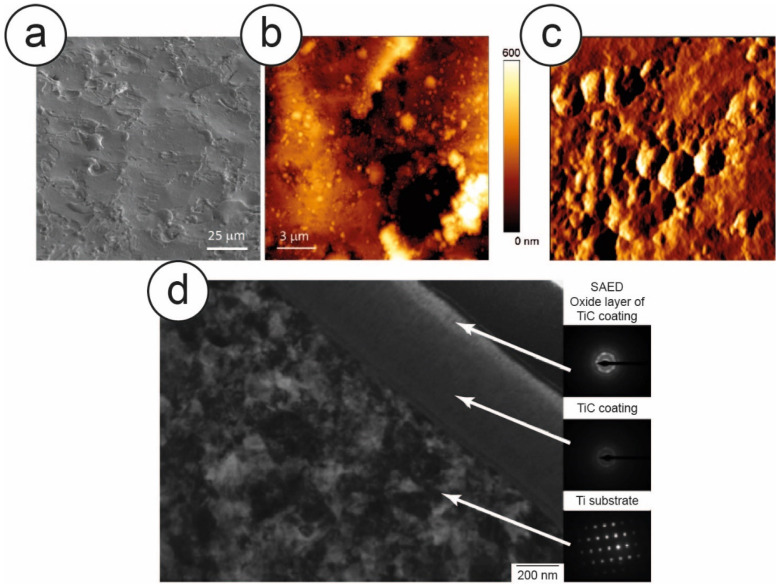
A collection of morphological images of the nanostructured TiC surface layer in a 900 W sample. (**a**) Scanning Electron Microscopy (SEM) image. (**b**) Large-scale topography and (**c**) high-resolution error signal Atomic Force Microscopy (AFM) images. (**d**) Analysis with Focused Ion Beam (FIB)/Transmission Electron Microscope (TEM)-Selected Area Electron Diffractometer (SAED) to determine the different layers of the film and the crystalline arrangement. The ion beam was used to reduce the sample to a thin lamella, which was then observed with TEM. Reprinted from [63]. Copyright (2015), with permission from Elsevier.

Zanoni et al. performed several tests using proliferation assays and MTT, to demonstrate that the TiC film deposited by IPPA did not produce any toxicity, and that the substrate biocompatibility was not altered by the IPPA treatment. At the same time, they were able to determine the ideal parameters to produce the highest stimulating effect on the cells [63]. As for the PLAD layers, the morphology of the film was then characterized by Longo et al., combining SEM and AFM imaging. (Figure 4a–c). The IPPA-deposited layer appeared rough and patchy, with defects homogeneously distributed on the entire area, without any spalls and with uniform roughness [64].

**Figure 5:**
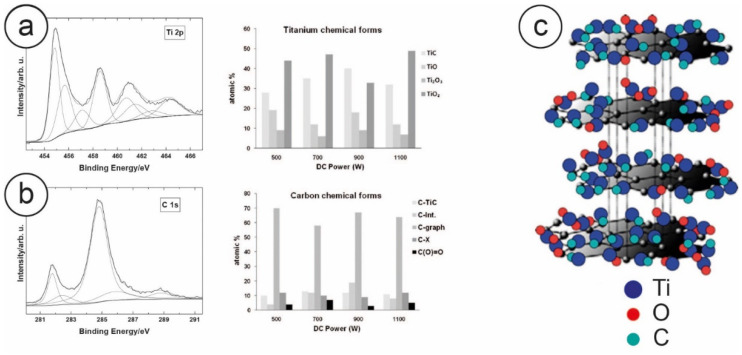
The distribution of the oxides in the layer was determined from the X-ray Photoelectron Spectroscopy (XPS) spectra of the film by investigating the Ti2p region, while the carbon species was obtained by studying the C1s spectra (panels **a** and **b**), both taken with Al kα monochromatized radiation, and the relative abundance histograms. This allows us to propose the reported pictorial model (**c**) of the TiC-enriched layer, where Ti oxides (TiO_2_), TiC and graphitic carbon are strictly connected to form a cluster as a consequence of the deposition conditions adopted. Reprinted from [63]. Copyright (2015), with permission from Elsevier.

By using a Focused Ion Beam (FIB) for micro and nano sample manufacturing, coupled with a TEM equipped with a Selected Area Electron Diffractometer (SAED) for the analysis of the crystalline structure of the sample, Zanoni and coworkers determined the thickness of the film, which was about 500 nm. (Figure 4d). By coupling it with a nanoindenter, they also performed nano and micro scratch tests, which revealed that this layer was well bound to the bulk titanium, showing a hardness (26.9 GPa) about five times that of titanium (4.4 GPa), and twice the elastic modulus (299 GPa) of Ti (129) [63,65]. In the same work, the authors also used XPS to determine the chemical composition of the layer, showing that the IPPA produced a film composed of 60% carbon, 15% TiC and 25% titanium oxides. (Figure 5a,b). Building on previous results [57] and combining XPS and Raman information, we can strongly suggest that the two major peaks of the C1s spectra, namely at 281.8 eV and at 284.8 eV, may be attributed to carbidic carbon and to graphitic carbon, respectively. The presence of graphitic carbon in particular was confirmed by Raman spectroscopy [63]. Indeed, due to the high similarity between the PLAD and the IPPA processes, this indicates that both processes form graphitic carbon in their respective layers, clustered with titanium carbide and titanium oxides, as depicted in Figure 5c.

#### 2.2.2. Cellular Adhesion

The processes through which the cells adhere to a substrate are very complex, and involve at least four steps which give way to the subsequent proliferation and differentiation of the cell: at first there is protein adsorption, followed by the formation of contact points between the cell and the substrate; next is the cellular attachment, and finally the spreading of the cell. The interactions between cells and substrate are dependent on both the substrate’s chemical structure [66,67] and on the surface’s physical properties, such as its local roughness [68,69], its wettability (which is determined by the contact angle that water droplets form with the dry surface) and its surface free energy. In particular, a contact angle smaller than 90° indicates a hydrophilic or partially hydrophilic surface (good wettability), while a contact angle greater than 90° defines a hydrophobic surface [70,71,72,73,74,75]. Lampin and coworkers showed that, for the best biocompatibility, the wettability of a surface should be about 70° [76].

Remarkably, Longo et al. showed that the water contact angle of uncoated titanium disks is 60.0° ± 2°, while the ones coated with the nanostructured film had an angle of 70.5° ± 2.3°, with a net increase in hydrophobicity of approximately 18%. Regarding the surface free energy, which was calculated according to the Van Oss–Chaudhury–Good method [74], a TiC-coated sample had values which were smaller than those of uncoated substrates, but this reduction was almost all concentrated in the reduction of the surface polar component, which was very low (γ_p_ = 0.13 mJ/m^2^), as was the acid fraction of the surface free energy [64]. The combination of the water contact angle, the decrease of surface free energy, and the large reduction of the polar and acidic fraction, leaving only the surface free energy basic fraction, indicates that the TiC-coated samples can favor osteoblast adhesion, since these cells are among the cells that react more strongly to the chemistry of the substrates. The coating can stimulate a profitable bidirectional cross-talk between cells and implants, enhancing the production of integrins, a group of membrane receptors that mediate the cellular adhesion to the extracellular matrix (ECM). These receptors are distributed on the cell membrane and can sense the specific chemical composition of the environment. They interact with paxillin, talin and other proteins that are part of the focal adhesion kinase complex, which induce changes in the cytoskeleton to respond to external stimuli, including the substrate properties [77,78,79]. In the particular case of osteoblasts, this is of particular importance, and ITGA3 is the gene which is upregulated by a positive adhesion to the substrate [80]. The interaction between the resulting dimer α3β1 and the integrins, transfers the chemical signal from the external environment, through the cell membrane, amplified by the cytoskeleton, to the cell nucleus, where adhesion, spread and cellular migration genes are activated, thus stimulating the growth processes and cellular differentiation [66,67,81,82,83].

There are several ways to evaluate directly the cellular adhesion strength [84]. Bulk experiments are the most commonly used methods, which involve growing the cells on the substrate, exposing them to a buffer which produces cell detachment, and monitoring the percentage of cells which are capable of maintaining their adhesion to the surface. By performing such analyses, Longo et al. showed that approximately 60% of the cells growing on untreated titanium surfaces had detached after this treatment, while in the case of the cells incubated on TiC-coated surfaces, only 35% were removed [64]. A second technique to perform such investigations exploits the high force sensitivity and the single cell capability of AFM: the Single Cell Force Spectroscopy [85,86,87]. These experiments showed that only 20 s are needed for the osteoblasts to form adhesion points towards the TiC substrates, and that the adhesion forces were much higher for the cell-TiC interactions than for the cell-Ti [64]. This is a clear indication that osteoblasts can evaluate very rapidly the chemical composition of the surface (around 20 s), and that, in the same timeframe, can activate the metabolic pathways which stimulate the substrate interaction. This is further indicated by the quantitative determination of the interaction forces.

#### 2.2.3. Effects of IPPA-Treated Substrate Topography and Chemistry on Osteoblasts Growth: Biochemistry, Immunofluorescence and Microscopy

As in the case of the PLAD films, to determine the stimulating activity of the IPPA films on osteoblasts, the toxicity of the layer and the cell adhesion, proliferation and gene expression must be characterized. In this case, Longo et al. showed, using the MTT viability test, that human osteoblasts grown for three days on the coated titanium disks had a 20% higher proliferation than those incubated on uncoated titanium disks. This difference was reduced to 10% after seven days. This indicated that the nanostructured layer produced a stimulating activity on osteoblasts [64].

To determine osteoblast viability and function, the presence of osteogenic differentiation factors, such as ALP and TGFβ1, as well as the gene expression of OC, COL1, PAX, ITGA-3, FHL1 and RUNX-2, are of paramount importance. As in the PLAD case, these factors were evaluated through enzyme-linked immunosorbent assay (ELISA) [88] and q-RT-PCR, showing a rapid upregulation effect of TiC. After a longer period of incubation, the increase in gene expression levels had disappeared for both Saos-2 and hOB, demonstrating that the effect of the chemistry of the nanostructured film had been exerted only at the early steps of the cell adhesion, and that the information on the chemistry of the environment had been transferred into the cell [64]. This was subsequently also performed using other primary cell lines, showing similar upregulation [89].

**Figure 6:**
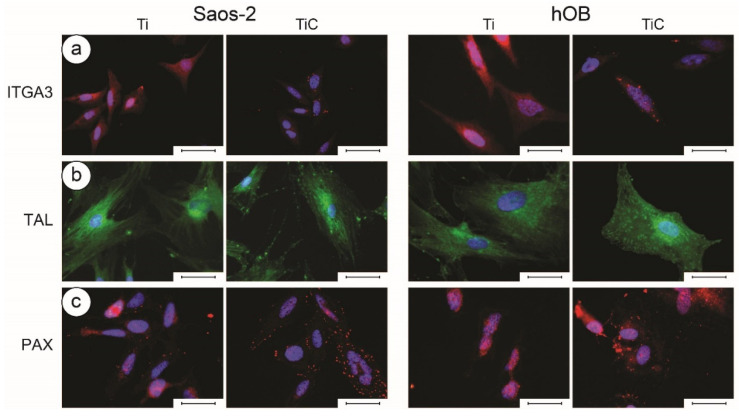
Immunofluorescence images of integrin α3β1, talin and paxillin in Saos-2 cells and in human primary osteoblasts (hOB). Panel (**a**): The Saos-2 (left panels) and the hOB cells (right panels) were grown for 96 h on glass slides coated with 10.5 nm of titanium or the nanostructured TiC layer, treated with primary monoclonal antibodies against integrin α3β1 (10 µg/mL). Panel (**b**): The cells were treated with primary monoclonal antibodies against talin (10 µg/mL). Panel (**c**): Cells were treated with primary monoclonal antibodies against paxillin (10 µg/mL). In all cases, the treatment was followed with Alexa Fluor 568 goat anti-mouse secondary antibodies, diluted 1:500, and the nuclei were stained with the organic dye DAPI. The images were collected with a magnification of 63× for ITGA and PAX, and of 100× for TAL, and the bar represents 100 µm. Obtained with permission from [64]. Copyright (2016), with permission from PLoS ONE.

**Figure 7:**
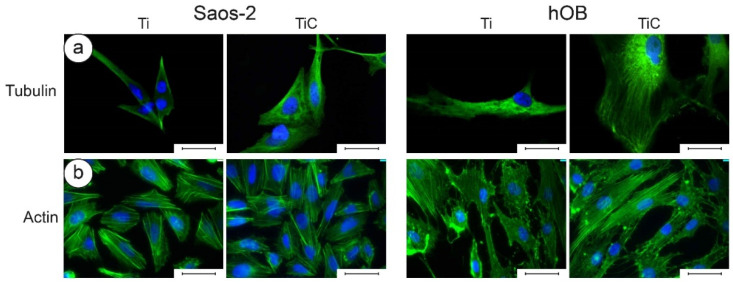
Immunofluorescence images of tubulin and actin in Saos-2 cells and in human primary osteoblasts. Panel (**a**): The Saos-2 (left panels) and the hOB cells (right panels) were grown for 96 h on glass slides coated with 10.5 nm of titanium or the nanostructured TiC layer, treated with primary monoclonal antibodies against tubulin (tubulin mouse monoclonal antibody 10 µg/mL) and Alexa Fluor 568 goat anti-mouse secondary antibodies, diluted 1:500. Panel (**b**): The cells were treated with Phalloidyn Alexa Fluor 488-conjugated, diluted 1:10. In all images, the nuclei were stained with DAPI. The images were collected with a magnification of 63×, and the bar represents 100 µm. Obtained with permission from [64]. Copyright (2016), with permission from PLoS ONE.

Since immunofluorescence requires optical microscopy imaging, the transparency of the substrates is fundamental. For these applications, glass slides coated with about 10 nm of film can be employed to simulate the full substrates. Indeed, in the case of IPPA deposition, XPS spectra confirmed that the chemical composition of these layers was compatible with that of the IPPA-coated discs. The coated glass slides allowed the transmission of 25% of the light, which was sufficient to perform fluorescence microscopy analyses. Longo et al. employed such substrates to perform a comparison between TiC- and Ti-coated glass slides. The cells grown on glass slides coated with the nanostructured film had a higher number of ITGA3 receptors (Figure 6a, red spots), TAL receptors (Figure 6b, green spots) and PAX receptors (Figure 6c, red spots). By exposing the cells to fluorescent dyes, the analysis showed that the tubulin was better distributed around the nucleus and in the cytoplasm, and that actin cytoskeleton was better defined in cells grown on TiC-coated glass slides. (Figure 7) [64]. These results demonstrated that the chemical and morphological information captured by receptors of integrin α3β1, talin and paxillin were transferred through the cytoskeleton to the synthetic apparatus of the cell, which, in the case of coated titanium disks, responded with a higher production of a cytoskeleton of better quality.

**Figure 8:**
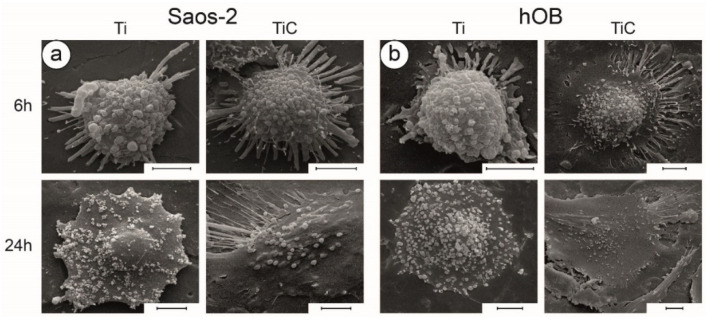
Investigation of cell morphology by SEM. Panel (**a**): SEM micrographs showing the morphology of Saos-2 cells grown for 6 h and 24 h on uncoated (Ti) and TiC-coated (TiC) titanium disks. Panel (**b**): similar analysis on hOB cells. The images reveal that both types of cells are richer in philopodia and lamellipodia, and better adhered to the substrate when grown either for 6 or 24 h on the TiC-coated titanium disks compared to the uncoated. In each figure, the bars represent 5 µm. Reprinted from [63]. Copyright (2015), with permission from Elsevier.

The combination of optical microscopy, AFM and SEM is the best way to fully characterize the morphology of cells [24,90]. Optical microscopy evidenced that several cells grown for 6 h on Ti coated disks had a rounded form, whereas several cells grown for the same time on TiC-coated disks had a more elongated form. This was confirmed by AFM, which revealed the presence of a higher number of filopodia on cells grown on the TiC-coated glasses and a lower number of filopodia on cells grown on the Ti coated glasses [64]. The SEM images showed similar morphologies, where, after 6 h of incubation, the Saos 2 cells (Figure 8a) and the hOB (Figure 8b) grown on Ti substrates exhibited a rounder form, with a smaller number of filopodia and lamellipodia. The differences were enhanced after longer incubation times (24 h), where the TiC layer produced more flat cells, more spread out and with longer cellular extensions (Figure 8, bottom panels) [64]. On the other hand, the osteoblasts incubated on untreated titanium substrates had fewer cellular extensions, and their shape was similar to that evidenced in the first phases of substrate adhesion.

#### 2.2.4. In Vivo Experiments on the IPPA Layer

All these morphological, biological and biochemical results concur to suggest how IPPA deposition can produce nanostructured layers which stimulate osteoblast adhesion, spreading, and overall cellular colonization.

**Figure 9:**
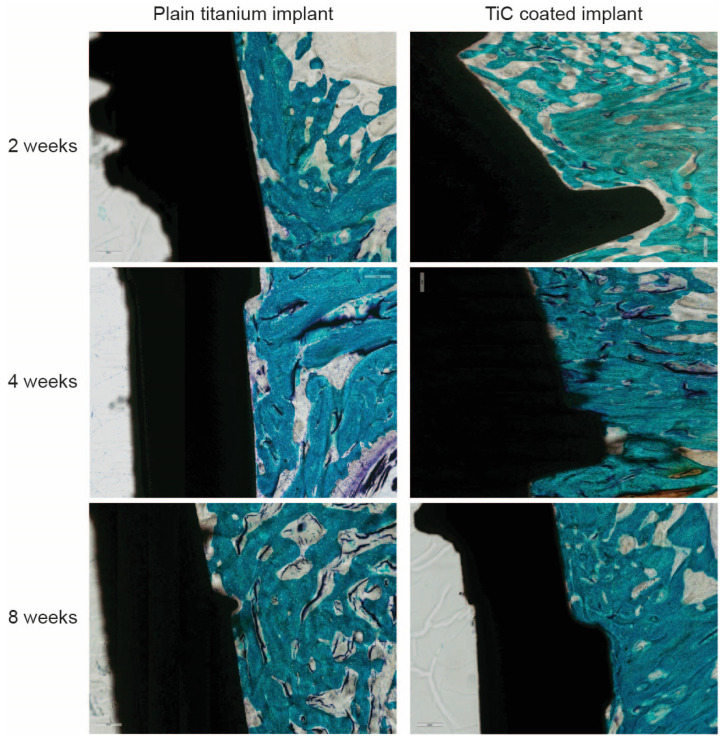
Histological images of the in vivo study of uncoated (Ti) and TiC-coated (TiC) titanium implants at 2, 4 and 8 weeks: 10× of magnification, scale bar is 200 µm. Toluidine blue and fast green staining. Reprinted from [91]. Copyright (2017), with permission from Elsevier.

Since all the data in vitro indicate how the mechanical, chemical and morphological properties of the hard, nanostructured TiC layer improve the osseointegration process, stimulating osteoblast proliferation, adhesion and activity, the subsequent step was to perform experiments similar to those already performed with the PLAD-coated implants in vivo. These results are detailed in Veronesi et al., where X-ray images, histological analyses and intravital fluorochrome experiments are combined to demonstrate the improved response of the bone formation near the TiC-coated implants, compared to the plain titanium ones [91] (Figure 9). All the analyses, including histomorphometric assays, histological evaluations, bone–implant contact measurements and Bone Ingrowth values of the bone formation around TiC implants were significantly higher than the cases in which bare Ti was implanted. Similarly, the mineral apposition rate and bone formation rate values were higher for the TiC-coated implanted material [91].

In conclusion, all the measured parameters in this in vivo study, even if on a limited number of animals, confirm the results obtained in vitro with the osteoblast cells, which all indicate that the coating with the nanostructure deposited by the IPPA technology has many beneficial effects, which, at the end, bestow a superior osseointegration efficiency on the TiC-coated implants when compared with the uncoated ones.

## 3. Conclusions

In this review, we have followed the biological itinerary that was used to demonstrate how TiC-coated implants produce increased osseointegration. We have shown how the deposition has been performed using IPPA technology, which has been demonstrated to form a nanostructure composed of graphitic carbon, a highly biocompatible material whose biocompatibility is enhanced by its binding to titanium oxides and titanium carbide. The stiffness of the nanostructure is particularly useful to protect the bulk of the implants, avoiding any further oxidation caused by the oxidizing environment of biological tissues.

Remarkably, the structural and chemical composition of the films deposited by the IPPA technology is very similar to that of the film deposited with other techniques that have been employed to coat different substrates with a TiC layer, including the PLAD technology, with the only difference being in their respective compactness, which is higher in the IPPA film.

In all cases, the morphology and chemistry of the TiC nanostructured coating caused the cells to initiate a profitable bidirectional cross-talk through their integrins, specifically activated by the chemistry of the environment, allowing the chemical environmental stimuli to rapidly reach the nucleus, inducing a rapid upregulation of bone turnover genes, increased cellular adhesion, spread, and migration, and overall stimulating the growth processes and cellular differentiation. The strength of the adhesion to the substrate, the formation of philopodia and lamellipodia, and the overall improved proliferation, adhesion and activity, indicate that TiC is capable of enhancing the osseointegration process. These results were confirmed by several works detailing experiments in vivo, using titanium implants coated with TiC to underline that the osseointegration process of the TiC-coated implants is more efficient than that of uncoated ones.

Recent studies have indicated that the stimulating effect of this nanostructured thin film is not limited to osteoblasts, but that TiC also provides improved cellular proliferation and adhesion on other cell types typically employed in research laboratories [89]. Since the thickness of the TiC layer can be regulated to be as small as 10 nm, this coating can be applied to glass slides to use in inverted optical microscopes and in fluorescence microscopes.

Furthermore, since the deposition with IPPA is inexpensive and easily implementable in an industrialized process, this could be proposed as a simple addition to the substrate preparation protocols, to improve their biocompatibility and osseointegration capability.
